# The preventive effect of atorvastatin on atrial fibrillation: a meta-analysis of randomized controlled trials

**DOI:** 10.1186/1471-2261-14-99

**Published:** 2014-08-13

**Authors:** Qian Yang, Xiaoyong Qi, Yingxiao Li

**Affiliations:** 1 Department of Cardiology, Hebei General Hospital, Shijiazhuang, Hebei, People’s Republic of China

**Keywords:** Atrial fibrillation, Atorvastatin, Meta-analysis

## Abstract

**Background:**

A number of clinical and experimental studies have investigated the effect of atorvastatin on atrial fibrillation (AF), but the results are equivocal. This meta-analysis was performed to evaluate whether atorvastatin can reduce the risk of AF in different populations.

**Methods:**

We searched PubMed, EMBASE and the Cochrane Database for all published studies that examined the effect of atorvastatin therapy on AF up to April 2014. A random effects model was used when there was substantial heterogeneity and a fixed effects model when there was negligible heterogeneity.

**Results:**

Eighteen published studies including 9952 patients with sinus rhythm were identified for inclusion in the analysis. Ten studies investigated primary prevention of AF by atorvastatin in patients without AF, seven studies investigated secondary prevention of atorvastatin in patients with AF, and one study investigated mixed populations of patients. Overall, atorvastatin was associated with a decreased risk of AF (odds ratio (OR) 0.51, 95% confidence interval (CI) 0.36–0.70, *P* < 0.0001). However, subgroup analyses showed that in the primary prevention subgroup (OR 0.55, 95% CI 0.38–0.81, *P* = 0.002), atorvastatin reduced the risk of new-onset AF in patients after coronary surgery (OR 0.44, 95% CI 0.29–0.68, *P* = 0.0002), but had no beneficial effect in patients without coronary surgery (OR 0.97, 95% CI 0.59–1.58, *P* = 0.89); in the secondary prevention subgroup, atorvastatin had no beneficial effect on AF recurrence in patients with electrical cardioversion (EC) (OR 0.57, 95% CI 0.25–1.32, *P* = 0.19) or without EC (OR 0.38, 95% CI 0.14–1.06, *P* = 0.06).

**Conclusions:**

This meta-analysis suggests that atorvastatin has an overall protective effect against AF. However, this preventive effect was not seen in all types of AF. Atorvastatin was significantly associated with a decreased risk of new-onset AF in patients after coronary surgery. Moreover, atorvastatin did not prove to exert a significant protective effect against the AF recurrences in both patients who had experienced sinus rhythm restoration by means of EC and those who had obtained cardioversion by means of drug therapy. Thus, further prospective studies are warranted.

## Background

Atrial fibrillation (AF) is the most common arrhythmia in clinical practice and is a major contributor to morbidity and mortality [1,2]. However, the mechanism of AF remains incompletely understood and treatment is not satisfactory. In recent years, an increasing number of studies have suggested that inflammation and oxidative stress contribute to atrial remodeling and play an important role in AF development [2-4]. It has been suggested that statin medications may be beneficial in protecting against AF, because of their anti-inflammatory and antioxidant properties [5]. Recent meta-analyses [6,7] of randomized controlled trials (RCTs) showed that statin therapy was significantly associated with a decreased risk of AF in different patient populations. Atorvastatin is a highly effective statin medication and is widely used clinically. It is the most studied statin in relation to AF [6,7], but the results are equivocal. We therefore performed this meta-analysis to evaluate whether atorvastatin can reduce the risk of AF in different populations.

## Methods

### Literature search and inclusion criteria

We searched PubMed, EMBASE and the Cochrane Database for all published studies that examined the effect of atorvastatin therapy on AF up to April 2014. We conducted text searches with the search terms “atorvastatin” and “atrial fibrillation”. We also manually searched references from selected clinical trials, recent meta-analyses and review articles.

We included studies that met the following specified criteria: (1) comparison of atorvastatin with placebo or control treatment, regardless of the background therapy; (2) randomized controlled human trials; (3) new-onset AF or recurrent AF in each group as an outcome.

### Data extraction and quality assessment

We extracted the following information from each study: (1) study population sample size and characteristics; (2) dose and duration of treatment; (3) outcome measures. One reviewer abstracted the data, and then the other checked the documentation. They finally reached an agreement on the data by consensus. We used the Jadad score [8] to assess the methodological quality of the included studies.

### Statistical analysis

We performed the statistical calculations with RevMan version 5.2 (The Cochrane Collaboration, Oxford, UK) and Stata version 12 (Stata Corp, College Station, TX, USA). We allocated the results of each study as dichotomous frequency data to evaluate the effect of atorvastatin on new-onset AF or recurrence of AF. We calculated the odds ratio (OR) and 95% confidence interval (CI) for new-onset or recurrent AF in each trial separately, and for combinations of studies according to fixed-effect and random-effect models. We used the chi-squared test to assess heterogeneity and *I*^2^ to quantify heterogeneity. If the chi-squared test *P*-value was > 0.10 and *I*^2^ was < 50%, we analyzed the data using a fixed-effect model (the Mantel–Haenszel method), otherwise we used a random-effect model [9,10]. If the heterogeneity was significant, we attempted to explain the differences based on the patient clinical characteristics of the included studies. The *P*-value threshold for statistical significance was set at 0.05 for the effect sizes. We also used Begg and Egger tests [11,12] to assess the presence of publication bias.

## Results

### Characteristics of the included studies

**Figure 1:**
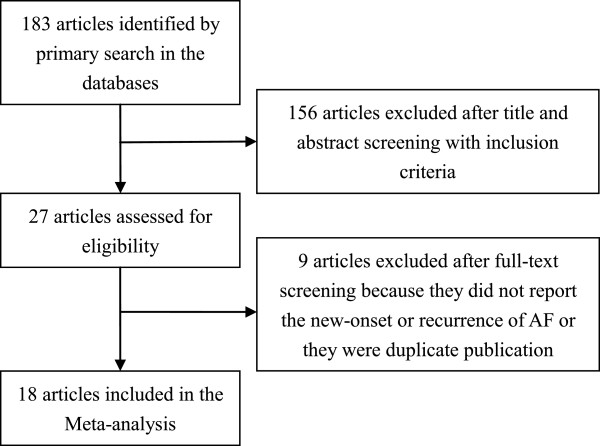
**Summary of the study selection and exclusion process.** AF: atrial fibrillation.

Eighteen published studies [13-30] including 9952 patients with sinus rhythm were identified for inclusion in the analysis. The process of study selection is summarized in Figure 1. These studies compared the use of atorvastatin vs. no statins on AF in various populations. Ten studies [15,17-19,21-24,26,28] investigated the use of atorvastatin in primary prevention of AF in patients after cardiac surgery (n = 8) [15,17,19,21-24,28] or implantation of a pacemaker (n = 1) [18], or in patients with a prior stroke or transient ischemic attack (n = 1) [26]. Seven studies [14,16,20,25,27,29,30] investigated the use of atorvastatin in secondary prevention of AF in patients who received electrical cardioversion (EC; n = 4) [16,20,25,27], catheter ablation (n = 2) [29,30] or pharmacological treatment (n = 1) [14]. The MIRACL trial [13] included both AF and non-AF patients, so MIRACL-1 and MIRACL-2 were used to represent the primary and secondary prevention subgroups. These studies were published between 2004 and 2013 and the sample sizes ranged from 40 to 4731. Intervention strategies of atorvastatin and documentation of AF were also variable. The studies received Jadad scores of 2 (n = 5) [16,18,21,27,30], 3 (n = 3) [14,19,24], 4 (n = 3) [15,23,29], or 5 (n = 7) [13,17,20,22,25,26,28] points. The characteristics of the studies included in the meta-analysis are shown in Table 1.

**Table 1:** Baseline characteristics of the studies included in the meta-analysis

**Study, year**	**n**	**Population**	**Dosage**	**Duration**	**Endpoint**	**Documentation of AF**	**Quality score**
MIRACL 2004 [13]	3087	Acute coronary syndrome	80 mg/d	16 weeks	New onset or recurrence of AF	Follow-up with ECG	5
Dernellis et al. 2005 [14]	80	Paroxysmal AF	20–40 mg/d	4–6 months	Recurrence of AF	48-h ambulatory ECG monitoring	3
Chello et al. 2006 [15]	40	Coronary bypass surgery	20 mg/d	3 weeks	New-onset AF after cardiac surgery	ECG monitoring	4
Ozaydin et al. 2006 [16]	48	Persistent AF and scheduled EC	10 mg/d	3 months	Recurrence of AF	24-h ambulatory ECG monitoring	2
ARMYDA-3 2006 [17]	200	Cardiac surgery	40 mg/d	30 days	New-onset AF after cardiac surgery	ECG monitoring after the operation, then follow-up with ECG	5
Tsai et al. 2008 [18]	106	Bradyarrhythmia and implantation of pacemaker	20 mg/d	1 year	AF/AHE ≥ 10 min	Pacemaker interrogation	2
Song et al. 2008 [19]	124	Off-pump coronary bypass surgery	20 mg/d	30 days	New-onset AF after cardiac surgery	ECG monitoring after the operation, then follow-up with ECG	3
Almroth et al. 2009 [20]	234	Persistent AF and scheduled EC	80 mg/d	30 days	Recurrence of AF	Follow-up with ECG	5
Melina et al. 2009 [21]	632	Coronary bypass surgery	40 mg/d	Not mentioned	New-onset AF after cardiac surgery	Not mentioned	2
Ji et al. 2009 [22]	140	Off-pump coronary bypass surgery	20 mg/d	13 days	New-onset AF after cardiac surgery	ECG monitoring or 12-lead ECG	5
Spadaccio et al. 2010 [23]	50	Coronary bypass surgery	20 mg/d	7 days	New-onset AF after cardiac surgery	ECG monitoring	4
Sun et al. 2011 [24]	100	Coronary bypass surgery	20 mg/d	14 days	New-onset AF after cardiac surgery	ECG monitoring	3
SToP AF 2011 [25]	64	Persistent AF and scheduled EC	80 mg/d	12 months	Recurrence of AF	Follow-up with ECG and 24-h ambulatory ECG monitoring	5
SPARCL 2011 [26]	4731	Prior stroke or transient ischemic attack	80 mg/d	4.8 years	New-onset AF	Follow-up with ECG	5
Demir et al. 2011 [27]	48	Persistent AF and scheduled EC	40 mg/d	2 months	Recurrence of AF	Weekly follow-up with ECG	2
Baran et al. 2012 [28]	60	Coronary bypass surgery	40 mg/d	30 days	New-onset AF after cardiac surgery	ECG monitoring after the operation, then follow-up with ECG	5
Suleiman et al. 2012 [29]	125	AF undergoing catheter ablation	80 mg/d	3 months	Recurrence of AF	Follow-up with ECG and 72-h ambulatory ECG monitoring	4
Jiang et al. 2013 [30]	101	AF undergoing catheter ablation	40 mg/d	6 months	Recurrence of AF	Not mentioned	2

AF: atrial fibrillation; AHE: Atrial high rate episode; ARMYDA-3: Atorvastatin for Reduction of MYocardial Dysrhythmia After cardiac surgery study; EC: Electrical cardioversion; ECG: Electrocardiogram; MIRACL: Myocardial Ischemia Reduction with Aggressive Cholesterol Lowering study; SPARCL: Stroke Prevention by Aggressive Reduction in Cholesterol Levels; SToP AF: statin therapy for the prevention of atrial fibrillation trial.

### Results of the meta-analysis

**Figure 2:**
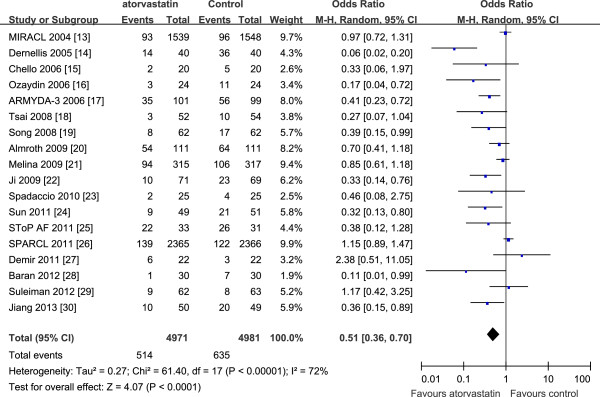
**Effect of atorvastatin on atrial fibrillation.** ARMYDA-3: atorvastatin for reduction of myocardial dysrhythmia after cardiac surgery study; CI: confidence interval; MIRACL: myocardial ischemia reduction with aggressive cholesterol lowering study; SPARCL: Stroke Prevention by Aggressive Reduction in Cholesterol Levels; SToP AF: statin therapy for the prevention of atrial fibrillation trial.

The meta-analysis of all included studies indicated that atorvastatin was effective for the prevention of AF (OR 0.51, 95% CI 0.36–0.70, *P* < 0.0001), but there was substantial heterogeneity (*P* < 0.00001, *I*^2^ = 72%). The results are shown in Figure 2.

### Subgroup analysis

**Figure 3:**
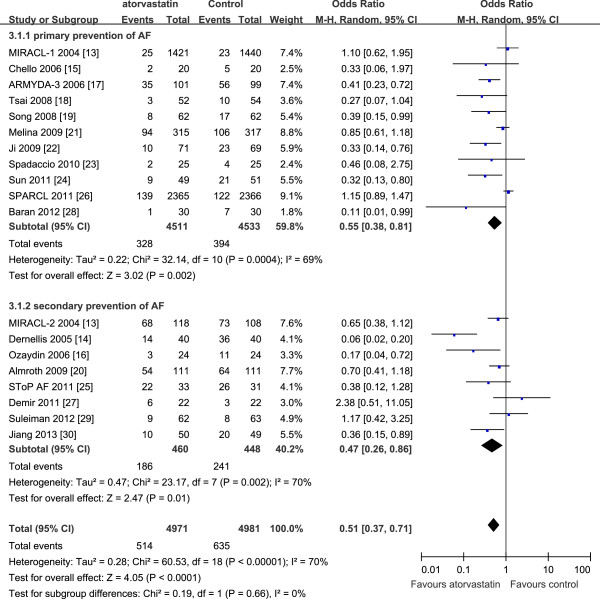
**Primary and secondary prevention of AF with atorvastatin.** AF: atrial fibrillation; ARMYDA-3: atorvastatin for reduction of myocardial dysrhythmia after cardiac surgery study; CI: confidence interval; MIRACL: myocardial ischemia reduction with aggressive cholesterol lowering study; SPARCL: Stroke Prevention by Aggressive Reduction in Cholesterol Levels; SToP AF: statin therapy for the prevention of atrial fibrillation trial.

A subgroup analysis was performed to evaluate the effect of atorvastatin on primary and secondary prevention of AF. The results showed that atorvastatin was effective for both primary prevention (OR 0.55, 95% CI 0.38–0.81, *P* = 0.002) and secondary prevention (OR 0.47, 95% CI 0.26–0.86, *P* = 0.01) of AF. However, the heterogeneity remained substantial (primary prevention: *P* = 0.0004, *I*^2^ = 69%; secondary prevention: *P* = 0.002, *I*^2^ = 70%) in both groups. The results are shown in Figure 3.

**Figure 4:**
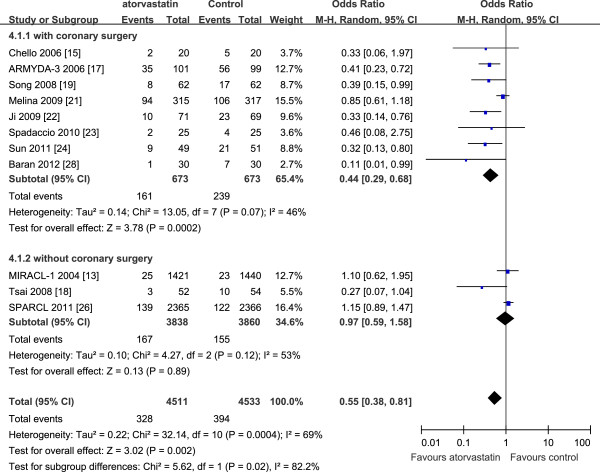
**Primary prevention of AF with atorvastatin in patients with or without coronary surgery.** AF: atrial fibrillation; ARMYDA-3: atorvastatin for reduction of myocardial dysrhythmia after cardiac surgery study; CI: confidence interval; MIRACL: myocardial ischemia reduction with aggressive cholesterol lowering study; SPARCL: Stroke Prevention by Aggressive Reduction in Cholesterol Levels.

Eleven trials [15,17-19,21-24,26,28] that investigated the use of atorvastatin in primary prevention of AF were divided into two subgroups on the basis of whether patients underwent coronary surgery. The results of subgroup analysis showed that atorvastatin was effective in reducing the risk of AF in patients after coronary surgery (OR 0.44, 95% CI 0.29–0.68, *P* = 0.0002), although there was heterogeneity (*P* = 0.07, *I*^2^ = 46%), but showed no beneficial effect in patients without coronary surgery (OR 0.97, 95% CI 0.59–1.58, *P* = 0.89), again showing heterogeneity (*P* = 0.12, *I*^2^ = 53%). The results are presented in Figure 4.

**Figure 5:**
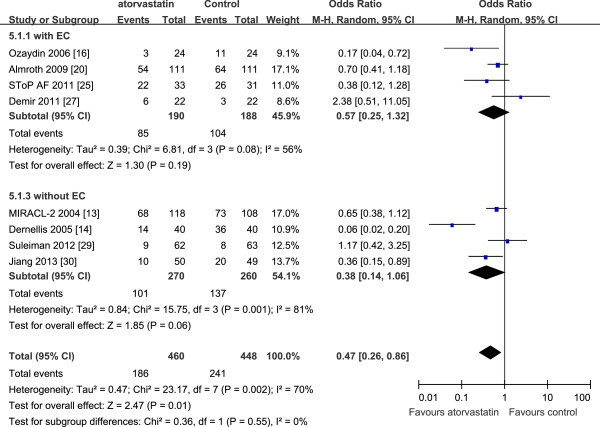
**Secondary prevention of AF with atorvastatin in patients with or without EC.** AF: atrial fibrillation; CI: confidence interval; EC: electrical cardioversion; MIRACL: myocardial ischemia reduction with aggressive cholesterol lowering study; SToP AF: statin therapy for the prevention of atrial fibrillation trial.

Eight trials [14,16,20,25,27,29,30] that investigated the use of atorvastatin in secondary prevention of AF were divided into two subgroups on the basis of whether patients underwent EC. The results of subgroup analysis showed that atorvastatin had no beneficial effect either in patients with EC (OR 0.57, 95% CI 0.25–1.32, *P* = 0.19) or without EC (OR 0.38, 95% CI 0.14–1.06, *P* = 0.06). The heterogeneity remained substantial (with EC: *P* = 0.08, *I*^2^ = 56%; without EC: *P* = 0.001, *I*^2^ = 81%) in both groups. The results are shown in Figure 5.

### Sensitivity analysis and publication bias

The results were similar when studies with Jadad score < 3 [16,18,21,27,30] were removed from the analysis (OR 0.49, 95% CI 0.32–0.73; *P* = 0.0004). These studies were also excluded when performing the subgroup analyses and no obvious change in effect size was detected. However, there was little heterogeneity in the coronary surgery subgroup (*P* = 0.96, *I*^2^ = 41%), so a fixed-effect model was used to analyze the data. The results are shown in Table 2.

**Table 2:** Sensitivity analysis

	**Number of trials**		**Effect size, odds ratio (95% CI)**	
**Variable**	**Total**	**After excluding 5 trials**^**#**^	**Total**	**After excluding 5 trials**^**#**^
Overall	18	13	0.51 (0.36 to 0.70)	0.49 (0.32 to 0.73)
Primary prevention	11	9	0.55 (0.38 to 0.81)	0.51 (0.31 to 0.84)
With coronary surgery	8	7	0.44 (0.29 to 0.68)	0.36 (0.25 to 0.51)
Without coronary surgery	3	2	0.97 (0.59 to 1.58)	1.14 (0.91 to 1.43)
Secondary prevention	8	5	0.47 (0.26 to 0.86)	0.46 (0.22 to 0.96)
With electrical cardioversion	4	2	0.57 (0.25 to 1.32)	0.63 (0.39 to 1.03)
Without electrical cardioversion	4	3	0.38 (0.14 to 1.06)	0.38 (0.09 to 1.62)

CI: confidence interval; ^#^Studies with Jadad score <3 were removed from the analysis.

Publication bias was evaluated using Begg’s and Egger’s tests when the subgroup analyses were performed. Publication bias was statistically insignificant in patients with EC (Begg, *P* = 1.00; Egger, *P* = 0.81), in patients without EC (Begg, *P* = 0.73; Egger, *P* = 0.45), in patients without coronary surgery (Begg, *P* = 0.30; Egger, *P* = 0.34), and in patients with coronary surgery when a trial with Jadad score < 3 was excluded [21] (Begg, *P* = 0.55; Egger, *P* = 0.18).

## Discussion

To the best of our knowledge, this is the first meta-analysis that excluded bias caused by different types of statins, and only assessed the efficacy of atorvastatin on the prevention of AF in different populations. The meta-analysis suggested that atorvastatin could protect against AF overall. However, subgroup analysis indicated that this preventive effect was not seen in all types of AF. Atorvastatin was significantly associated with a decreased risk of new-onset AF in patients after coronary surgery, while atorvastatin did not prove to exert a significant protective effect against the AF recurrences in both patients who had experienced sinus rhythm restoration by means of EC and those who had obtained cardioversion by means of drug therapy.

Atorvastatin is widely used in clinical practice, and more clinical studies related to AF have been carried out with atorvastatin than with other statins. In the latest meta-analysis [7], 28 RCTs were included to evaluate the preventive effect of statin therapy on AF. Of the 28 trials, atorvastatin was studied in 16, indicating that atorvastatin has the most evidenced-medicine data on AF. The meta-analysis [7] included all types of statins and suggested that statin therapy reduced AF significantly, with an OR of 0.69 (95% CI 0.57–0.83). The present meta-analysis suggests that atorvastatin is a highly effective statin medication in reducing AF, with an overall OR of 0.51 (95% CI 0.36–0.70). Rosuvastatin is another highly effective statin, but only limited data are available about its potential effect on AF. A previous meta-analysis of rosuvastatin [31] only included four RCTs and showed that it reduced the risk of AF by 30% (relative risk 0.70, 95% CI 0.54–0.91).

The preventive effect of atorvastatin against AF did not appear to be dose-dependent, as the trials that used a high dose of atorvastatin (80 mg/day) [13,20,25,26,29] did not have lower ORs. Trials with different doses of atorvastatin were limited and with significant heterogeneity in the study populations, so subgroup analysis according to different doses was not performed in our meta-analysis. A previous meta-analysis [32] even came to the conclusion that the preventive effect of atorvastatin on AF may be more significant at a lower dose (10–40 mg/day), while a recent study [33] on patients undergoing cardiac surgery suggested that the preventive effect of atorvastatin on postoperative AF was not dose-dependent.

In our meta-analysis, subgroup analyses were performed to evaluate the effect of atorvastatin on AF in different populations. Atorvastatin showed less atrial antiarrhythmic properties than expected in different populations, especially in the prevention of AF recurrence. In the primary prevention subgroup, the results showed that atorvastatin was significantly associated with a decreased risk of new-onset AF in patients after coronary surgery (Figure 4), which was in accord with other recent meta-analyses [7,34]. However, atorvastatin did not appear to protect against new-onset AF in patients who did not undergo coronary surgery (Figure 4). However, as only three trials were included in this subgroup and there was significant heterogeneity among them, it is inappropriate to come to the conclusion that atorvastatin was ineffective in this population. A recent meta-analysis [7] included nine RCTs and also found a negative result, which the authors suggested may be attributed to a relatively short follow-up time. In our secondary prevention subgroup, when trials were divided into two groups of patients with or without EC, the results showed that atorvastatin had no beneficial effect on the recurrence of AF in either group (Figure 5). These results contrasted with previous meta-analyses [6,35], which suggested that statin therapy significantly decreased the risk of AF recurrence in patients with or without EC. In our subgroup analysis, because only four trials were included in each subgroup and the study population of each study was relatively small, it is inappropriate to come to the conclusion that atorvastatin is ineffective in the secondary prevention of AF, and further investigation is required.

At present, an increasing amount of evidence suggests that inflammation and oxidative stress may play important roles in the pathogenesis and perpetuation of AF [2,36,37]. The mechanism of action of statins in the treatment of AF is suggested to be related to their anti-inflammatory and antioxidant properties [4,38]. The capacity of statins to reduce inflammation is relatively well established. Eight trials [14-17,22,24,25,28] in our meta-analysis proved that atorvastatin could reduce inflammatory biomarkers, especially in patients after cardiac surgery [15,17,22,24,28] when there was obvious acute inflammation. Furthermore, the nicotinamide adenine dinucleotide phosphate oxidase (NOX) is considered to be a very important cellular source of reactive oxygen species (ROS) in the human body, while excessive production of ROS is likely involved in the structural and electrical remodeling of the heart, and contributes to atrial fibrosis and AF [39]. Atorvastatin could inhibit ROS generation via downregulation of NOX2, thus protecting against AF [40]. In recent years, myeloperoxidase (MPO), a heme enzyme abundantly expressed by neutrophils, was shown to be a crucial prerequisite for atrial fibrosis and AF [2,41]. Statins could strongly inhibit MPO mRNA expression in human and murine monocyte-macrophages, and consequently reduce MPO protein and enzyme activity [42]. These may be the underlying mechanisms of statin therapy on AF and deserve further investigation.

This meta-analysis indicated that patients who underwent coronary surgery derived more benefit from atorvastatin in the prevention of AF than other populations. The reasons can be summarized as follows. The postoperative course of a coronary surgery is characterized by severe inflammation and oxidative stress resulting from extracorporeal circulation and surgical manipulation of the epicardial coronary vessels. Thus the anti-inflammatory and antioxidant effects of atorvastatin as mentioned above may account for its beneficial effects in these patients. AF after coronary surgery is mainly caused by elevations in atrial pressure, autonomic nervous system imbalance, myocardial ischemic damage and so on [43], thus it can regress over time with surgery recovery. However, non-postoperative AF, especially recurrent AF, is often caused by atrial remodeling such as atrial dilation and fibrosis [44]. This remodeling encompasses changes in electrical, contractile, and structural properties of the atria and may be less responsive to atorvastatin compared with postoperative AF.

This meta-analysis has the following limitations. Only published RCTs were included in our meta-analysis, so publication bias was unavoidable. Although subgroup analyses were performed according to different populations, heterogeneity still existed among the trials, such as sample size, the dose and duration of atorvastatin treatment, and documentation of AF. The trials were limited according to the numbers in some subgroups, so the results were less persuasive.

## Conclusions

The meta-analysis suggested that atorvastatin has an overall protective effect against AF. However, subgroup analysis indicated that this preventive effect was not seen in all types of AF. Atorvastatin was significantly associated with a decreased risk of new-onset AF in patients after coronary surgery. Moreover, atorvastatin did not prove to exert a significant protective effect against the AF recurrences in both patients who had experienced sinus rhythm restoration by means of EC and those who had obtained cardioversion by means of drug therapy. Thus, more large-scale prospective RCTs are required to investigate whether atorvastatin is an effective medication for the prevention of AF in different populations.

## Abbreviations

AF: Atrial fibrillation; RCTs: Randomized clinical trials; EC: Electrical cardioversion; MIRACL: Myocardial Ischemia Reduction with Aggressive Cholesterol Lowering study; ARMYDA-3: Atorvastatin for Reduction of MYocardial Dysrhythmia After cardiac surgery study; SToP AF: Statin therapy for the prevention of atrial fibrillation trial; SPARCL: Stroke Prevention by Aggressive Reduction in Cholesterol Levels; NOX: Nicotinamide adenine dinucleotide phosphate oxidase; ROS: Reactive oxygen species; MPO: Myeloperoxidase.

## Competing interests

The authors declare that they have no competing interests.

## Authors’ contributions

QY conceived the study, participated in the design, collected the data, performed statistical analyses and drafted the manuscript. XY conceived the study, collected the data, and helped to draft the manuscript. YL performed statistical analyses and helped to draft the manuscript. All authors read and approved the final manuscript.

## Pre-publication history

The pre-publication history for this paper can be accessed here:

http://www.biomedcentral.com/1471-2261/14/99/prepub
